# Beta-Arrestins and Receptor Signaling in the Vascular Endothelium

**DOI:** 10.3390/biom11010009

**Published:** 2020-12-23

**Authors:** Claudia Lee, Gayathri Viswanathan, Issac Choi, Chanpreet Jassal, Taylor Kohlmann, Sudarshan Rajagopal

**Affiliations:** 1 Department of Biochemistry, School of Medicine, Duke University, Durham, NC 27710, USA; claudia.lee@duke.edu; 2 Medical Center, Department of Medicine, Division of Cardiology, Duke University, Durham, NC 27710, USA; gayathri.viswanathan@duke.edu (G.V.); issac.choi@duke.edu (I.C.); 3 College of Arts and Sciences, The University of North Carolina at Chapel Hill, Chapel Hill, NC 27599, USA; cjassal@live.unc.edu; 4 Trinity College of Arts and Sciences, Duke University, Durham, NC 27708, USA; taylor.kohlmann@duke.edu

**Keywords:** beta-arrestin, G protein-coupled receptor, chemokine receptors, receptor tyrosine kinases, receptor serine-threonine kinases, vascular endothelial growth factor receptor (VEGFR), type II bone morphogenetic protein receptor (BMPR-II)

## Abstract

The vascular endothelium is the innermost layer of blood vessels and is a key regulator of vascular tone. Endothelial function is controlled by receptor signaling through G protein-coupled receptors, receptor tyrosine kinases and receptor serine-threonine kinases. The β-arrestins, multifunctional adapter proteins, have the potential to regulate all of these receptor families, although it is unclear as to whether they serve to integrate signaling across all of these different axes. Notably, the β-arrestins have been shown to regulate signaling by a number of receptors important in endothelial function, such as chemokine receptors and receptors for vasoactive substances such as angiotensin II, endothelin-1 and prostaglandins. β-arrestin-mediated signaling pathways have been shown to play central roles in pathways that control vasodilation, cell proliferation, migration, and immune function. At this time, the physiological impact of this signaling has not been studied in detail, but a deeper understanding of it could lead to the development of novel therapies for the treatment of vascular disease.

## 1. The Vascular Endothelium

Endothelial cells are a key modulator for vascular homeostasis. Comprising the innermost layer within blood vessels, these cells act not only as a barrier between tissue and blood moieties, but also a regulator of vascular tone based off of physical and chemical signaling inputs from the environment [1,2]. A major component of vascular tone regulation relies on nitric oxide (NO) based pathways in which diffusion of NO to vascular smooth muscle cells, leads to cGMP production leading to vasodilation [1]. This production of NO by endothelial NO synthase (eNOS) is regulated via several signals such as shear stress, bradykinin, vascular endothelial growth factor (VEGF) during hypoxia, and serotonin from platelet aggregation [1]. Other NO-independent modulators of vascular tone include prostacyclin, as well as vasoconstrictor substances such as endothelin-1 (ET-1), vascoconstrictor prostanoids, and angiotensin II (AngII).

In the normal vasculature, NO maintains the vascular wall in a dormant state through inhibition of inflammation, cell proliferation, and thrombosis. This NO-dominant phenotype is believed to maintained via laminar shear stress and is assisted via S-nitrosylation of cysteine residues in proteins such as NFκB and other cell cycle proteins [3]. Dysregulation of endothelial cells is typically marked by switch from a NO-dominant silencing of cellular processes, to redox-based signaling. In this switch, reactive oxygen species (ROS) lead to the generation of hydrogen peroxide (generated by superoxide dismutase). The peroxide, similar to NO, can spread through the cell and alter proteins via their cysteine groups. Doing so switches endothelial cells to a more a proliferative, proinflammatory, and prothrombotic state [3].This state is further exacerbated by the increased expression of specific chemokines and chemokine receptors [4]. The resulting changes are thought to contribute to several vascular diseases including atherosclerosis, hypertension, cardiac failure, as well as microvascular dysfunction in diabetes [2,3].

A central component to endothelial function is the receptors which respond to the large variety of environmental signals such as hormones, other cells within the vasculature (i.e smooth muscle cells), neurotransmitters, pericytes, cytokines and oxygen tension. These include but are not limited to G protein-coupled receptors (GPCRs), receptor tyrosine kinases (RTKs), and receptor serine-threonine kinases (RS/TKs). Regulating their downstream signaling, β-arrestins have emerged as a crucial effector for several of these receptors.

In this review article, we will focus on the roles of β-arrestin as a key partner in endothelial cell mediated signaling and function. We will summarize recent findings of major signaling pathways mediated by β-arrestin within chemokine receptors, as well as other pertinent GPCRs, RTKs and RS/TKs. Furthermore, we will discuss the impact of these on vascular endothelial cell function and their application to potential therapeutics for vascular disease.

## 2. β-Arrestins as a Common Signaling Node for Transmembrane Receptors

**Figure 1:**
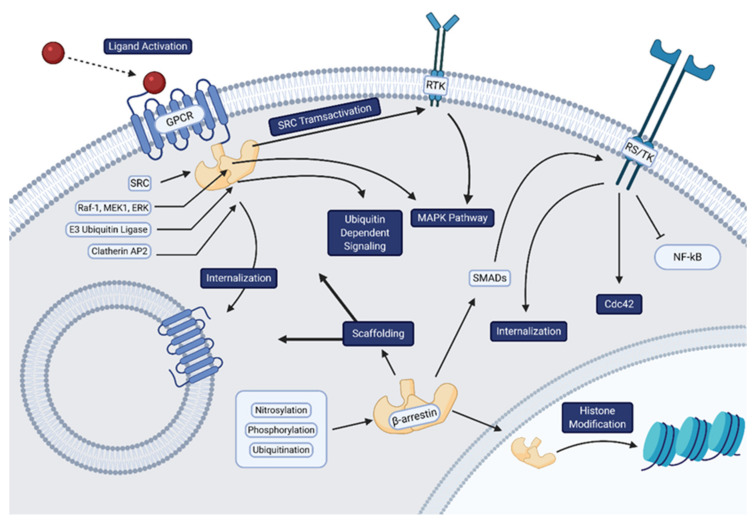
Overview of β-arrestin-mediated signaling. β-arrestins orchestrate a variety of cellular processes in addition to their originally discovered function of “arresting” GPCR signaling. These direct effects on membrane receptors such as internalization of GPCRs and transactivation of RTK and RS/TKs. β-arrestins can also promote their own signaling via scaffolding of several proteins (i.e MAPKs, Src, and clathrin), undergo various modifications such as ubiquitination, and influence epigenetic modifications. Created with BioRender.com.

The β-arrestins (β-arrestin1 and 2) are intracellular adapter proteins best known for their regulation of GPCR signaling and can perform a wide array of functions in the cell (Figure 1) [5]. With 78% sequence homology [6], the two β-arrestin isoforms have highly conserved structures consisting of two domains that pivot on an inter-domain hinge [7,8].They are predominantly found in the cytoplasm, while β-arrestin1 can also be found in the nucleus due to its nuclear localization signal [9]. Originally discovered to inhibit or “arrest” GPCR β-arrestins are now understood to have a complex role in regulated to varied array of cellular functions [10]. As with many other proteins, post translational modifications play a crucial role in dictating signaling and trafficking of β-arrestins. Both isoforms are constitutively phosphorylated (Ser412 for β-arrestin1, Ser361 and Thr383 for β-arrestin2) with dephosphorylation of the two β-arrestins controlling differential trafficking, desensitization kinetics, and recycling of receptors [11]. β-arrestin ubiquitination has been shown to result in sustained β-arrestin-GPCR interaction, prolonging MAP kinase (MAPK) activity [12]. β-arrestins also undergo various other post-translational modifications such as S-nitrosylation [13]. However, it is unclear as to how these modifications affect their signaling and functionality.

Though β-arrestins are classically understood to be a part of the machinery required for GPCR trafficking, it is now appreciated that they also act as signal transducers and transactivators for GPCRs and RTKs (Figure 1) [14]. After GPCR activation by a ligand, the receptor is phosphorylated, typically via G protein Receptor Kinases (GRKs), leading to β-arrestin recruitment [10,15,16,17]. Recruited β-arrestins can act as a scaffold that links the receptor and the machinery for internalization via clathrin and AP2 (Figure 1) [18,19]. Though some GPCRs do not require β-arrestin for their internalization, it has been shown that they do require them for recycling [20]. In addition to sterically inhibiting G proteins interactions within active receptors, β-arrestins also have the capability to initiate distinct signaling patterns [21]. β-arrestins have been shown to scaffold Raf-1, MEK1, and ERK (Figure 1) [22]. β-arrestins have also been shown to modulate ubiquitin dependent signaling by acting as an adaptor for E3 Ubiquitin ligase [23]. Counterintuitively, β-arrestins have also been shown to promote endosomal G protein signaling [24], specifically by the parathyroid hormone receptor [25] and the V2 Vasopressin receptor [26]. Nuclear β-arrestin1 is also able to directly influence epigenetic modifications by interacting with histone acetylases and deacetylases, influencing chromatin structures [26,27,28].

An additional level of complexity in β-arrestin signaling stems from the function divergence of the two β-arrestin isoforms [29]. In some cases, one isoform predominates certain downstream signaling events. For example, β-arrestin2 at the CXC Chemokine Receptor 4 and Atypical Chemokine Receptor 3 (ACKR3), predominantly mediates ERK activation and CCL12 accumulation respectively [30,31]. In other cases, these isoforms can have reciprocal effects on the same target. Reduction of β-arrestin 2 leads to decreased ERK activation at the Angiotensin II type I receptor (AT1R), whereas knockdown of β-arrestin1 augments ERK activation [32]. β-arrestin2 plays a predominant. The differential signaling between β-arrestin isoforms have also been noted in to play roles in the vasculature and disease. Mouse models of myocardial infarction have shown β-arrestin2 to offer protective effects via inflammation suppression under the β2AR whereas β-arrestin1 negatively effects recovery [33,34]. In vascular smooth muscle cells, β-arrestin2 signaling under the AT1R receptor leads to increased hypertrophy and hyperplasia in comparison to β-arrestin1 which decreases these effects [35]. β-arrestin2 in vascular endothelial cells and platelets.

Studies have implicated β-arrestins in endothelial and vascular function. For example, β-arrestin are reported to interact with caveolin-1, an abundant membrane protein in endothelial known present within caveolae, influence several endothelial cell functions, such as a membrane protein endocytosis of GPCRs and modulation of intracellular signaling events, extracellular matrix organization, and mechanotransduction [36]. The β-arrestin-caveolin complex is suggested to modulate NO production pathways in response to mechanotransduction via phosphorylation of Akt and eNOS [37]. β-arrestin knockout mice have been displayed to lead to disfunction in eNOS activity via GPCR kinase interactor 1 (GIT1) in sinusoidal endothelial cells leading to increased portal hypertension upon liver injury [38]. We will further discuss the specific roles β-arrestin plays in downstream signaling events under select receptors in the following sections. As highlighted before, we would like to emphasize these receptors often have multiple aspects of signaling, such as the heterotrimeric G proteins with many GPCRs, and the unaddressed influences of these factors are often a caveat of β-arrestin signaling studies.

## 3. β-Arrestin-Mediated GPCR Signaling in the Endothelium

In addition to producing a broad range of chemokines in response to proinflammatory stimuli, endothelial cells also express a variety of chemokine receptors [4]. Chemokines are organized into four subgroups via the arrangement of the first two of four conserved cysteine residues within the N-terminus (CC, CXC, CX3C, and C) [39]. Increased expression of these chemokines and chemokine receptors in response to various factors such as hypoxia, toxins, and shear stress play a major role in endothelial cell physiology and dysfunction. The accumulation of chemokines and their receptors often lead to proangiogenic and proinflammatory signals, contributing to diseased states such as cancer, atherosclerosis, pulmonary arterial hypertension (PAH), as well as autoimmune diseases [3,4]. β-arrestins also regulate signaling of receptors for vasoactive mediators, such as angiotensin II (AngII), endothelin-1 (ET-1) and prostaglandins. β-arrestins play central roles in regulating their signaling; for example, a number of GPCRs have been shown to activate eNOS, a key regulator of endothelial function, via β-arrestin2 [29]. We review some of the data focused on chemokine receptors and other GPCRs below (summarized in Table 1).

### 3.1. C-X-C Chemokine Receptor Type 3

Within endothelial cells, C-X-C chemokine receptor type 3 (CXCR3) serves as a prime regulator for pro-angiogenic and anti-angiogenic signals. CXCR3 is expressed as 3 reported splice variants (CXCR3A, CXCR3B, and CXCR3Alt) and primarily binds to the ligands CXCL4, CXCL9, CXCL10, CXCL11. CXCR3 activation by CXCL10 promotes cAMP production and protein kinase a (PKA) activation to inhibit VEGF-mediated endothelial tube formation [30]. CXCL10 also promotes p38/FAK signaling to induce migration as well as angiostatic activity via p38 [31,32]. Like most GPCRs, CXCR3 can signal via both G protein and β-arrestin pathways [33]. Several studies have demonstrated changes in β-arrestin signaling downstream between the splice variants, as well as between the different chemokine ligands at the predominant receptor isoform (CXCR3A) [34,35]. CXCR3B is primarily expressed on microvascular endothelial cells and is found to be more “arrestin-biased” than CXCR3A given β-arrestin is preferentially recruited in absence of chemokines [34,35]. In comparison to CXCR3A, which utilizes GRK2/3 and GRK5/6 to mediate β-arrestin2 recruitment, CXCR3B utilizes GRK2/3 [34]. CXCR3B activation via CXCL10 and CXCL4 results in antiproliferative and antimigratory response compared to CXCR3A within microvascular endothelial cells [36]. Although β-arrestin is previously reported to have a role in endothelial cell functionality via the FAK and MAPK pathways under other receptors, it is unclear whether this occurs downstream of activated endothelial cell CXCR3 as well [37].

### 3.2. C-X-C Chemokine Receptor Type 4

C-X-C chemokine receptor type 4 (CXCR4) has long been implicated in promotion of angiogenesis through its primary cognate ligand, CXCL12 [38,61,62]. CXCR4 activation induces a variety of secondary effector signaling such as MAPK/ERK, phosphoinositide 3-kinase (PI3K)/Akt, and Wnt/β-catenin pathways [61,63]. PI3K/Akt activation by CXCR4 in particular has been associated with increased expression of the pro-angiogenic factor VEGF [61,62]. While G proteins have been reported to promote many of CXCR4 downstream signaling functions, β-arrestin have been reported as a primarily negative regulator for CXCR4. Upon stimulation, CXCR4 is phosphorylated by various kinases, including GRK2, GRK3, GRK5, and GRK6. Phosphorylation at these key sites, two of which are Ser346 and 347, lead to β-arrestin recruitment and subsequent receptor desensitization and internalization [64,65]. Intriguingly, differential phosphorylation of CXCR4 in HEK293 cells by GRKs have been suggested to promote recruitment of particular β-arrestin isoforms and as a result, lead to differential downstream signaling [65]. GRK2, GRK6 phosphorylation of CXCR4 in HEK293 cells is associated with β-arrestin2 recruitment and negative regulation of calcium via receptor desensitization occurs primarily via, whereas GRK3, GRK6 phosphorylation is associated with β-arrestin1 positive regulation of ERK1/2 activation [65]. β-arrestin2 phosphorylation via ERK1/2 has also been shown to increase the intracellular distribution of the receptor, ultimately leading to decreased CXCR4 signaling [66]. In addition, endocytosis has been shown to be required for Akt activation by CXCR4, however it is unclear whether β-arrestins have a role in this process [62]. Heterodimerization of CXCR4 can impact β-arrestin signaling in other receptors within endothelial cells. CXCR4 interaction with protease-activated receptor 1 (PAR1) in human primary pulmonary endothelial cells reduces β-arrestin1 signaling to promote protective effects during endothelial barrier impairment from thrombin [40,41].

### 3.3. Atyptical Chemokine Receptor 3

Atypical chemokine receptor 3 (ACKR3, also known as CXCR7 or RDC-1) is co-expressed with CXCR4 in diverse cell types. These include vascular smooth muscle cell (VSMCs) [67] and vascular endothelial cells [68]. ACKR3 is thought to signal primarily through β-arrestin pathways, though underlying mechanisms are relatively unclear. ACKR3 is known to be phosphorylated via GRK2 to promote receptor endocytosis, however it is unknown which sites in particular are phosphorylated to promote β-arrestin recruitment [69,70]. ACKR3 activates ERKs or Akt [33,34,42], however, other data has suggested that this signaling may not depend on β-arrestins and is linked to signaling through other effectors that are sensitive to receptor phosphorylation [69,71]. CXCL12-stimulated ACKR3 acts as a functional receptor to activate Akt for angiogenesis in HUVECs [72]. During inflammatory conditions, both leukocytes and endothelial cells increase ACKR3 expression [73]. ACKR3 is also prominently expressed in a wide range of tumors both within the tumor cells and by cells of the tumor vasculature [74]. After stimulation with a small molecule ligand CCX771, ACKR3 has been shown to promote β-arrestin2-mediated signaling resulting in transendothelial migration of tumor cells. However, unlike other chemokine receptors, this occurred in the absence of classical GPCR-associated Ca2+ mobilization [43]. In addition, ACKR3 is induced in brain microvascular endothelial cells during experimental inflammatory conditions, such as permanent middle cerebral artery occlusion and experimental autoimmune encephalomyelitis (EAE) and promotes leukocyte extravasation by enhancing leukocyte adhesion to the endothelial surface [44].

### 3.4. The Angiotensin II Type 1 Receptor

The angiotensin II type 1 receptor (AT1R) is important in the regulation of vascular tone, and in proliferation and chemotaxis of vascular smooth muscle cells (VSMCs) [45]. Stimulation with AngII, leads to signaling mediated by both G protein and β-arrestins [45]. β-arrestin2 mediates the pathway responsible for late and prolonged ERK activation, which is important in promotion of cell survival pathways, such as Akt phosphorylation, and in the proliferation of VSMCs by regulating processes such as DNA synthesis [45,46]. This same pathway can be activated in the absence of G protein signaling by mechanical stress [46]. Specifically, mechanical stress causes the AT1R to enter an active conformation, in which it undergoes phosphorylation by GRK5 and GRK6, recruiting β-arrestin to the receptor [46]. Notably, double electron-electron resonance spectroscopy has indicated that when mediating β-arrestin-biased signaling, the AT1R is stabilized in a distinctly different conformation than when stimulated by AngII or G protein-biased ligands [75]. β-arrestin recruitment to the AT1R facilitates recruitment of factors such as Src kinase, which phosphorylates EGFR’s Tyr-845, and heparin-binding EGF to the receptor, allowing for EGFR transactivation, which facilitates ERK activation [45].

### 3.5. The Apelin Receptor

The apelin receptor (APJ) is chiefly found in the endocardium of the heart and the endothelium of the vasculature [76]. It is important in the mediation of migration and proliferation necessary for cardiovascular development as well as in the regulation of vascular tone. Specifically, APJ is important in NO-dependent and -independent vasodilatation to diminish vasoconstriction induced by AngII via the AT1R [47,76]. APJ binds a number of endogenous ligands, including apelin (and its derivatives) and the peptide Elabela/Toddler which commonly exert a G protein bias [47]. Lack of vasodilatation when APJ was stimulated with a synthetic G protein biased ligand, apelin-17, suggests that β-arrestin may mediate the pathways responsible for counteracting vasoconstriction elicited by AngII [47]. Additionally, in response to mechanical stress, APJ recruits β-arrestin, but not G proteins, resulting in hypertrophy and receptor internalization [47]. The residues Phe 257, Trp 263, and Ser 348 in the human APJ receptor have been determined necessary for this G protein-independent signaling via GRKs and β-arrestin [47].

### 3.6. β2 Adrenergic Receptor

The β2-adrenergic receptor (β2AR) is important in the regulation of vascular tone [77]. ^19^F-NMR examining the active conformation of the β2AR has shown that full agonists (such as isoproterenol) produce significant changes in the conformations of helices VI and VII, while β-arrestin-biased agonists (such as carvedilol and isoetharine) only impact helix VII; this suggests a structural basis for a separate, but parallel, nature of G protein and β-arrestin signaling at the β2AR [78]. Additionally, earlier studies have suggested that differential phosphorylation at the β2AR by GRK2 and GRK6, similar to that of CXCR4, lead to distinct β-arrestin signaling where GRK2 primarily influenced receptor internalization and GRK6 sites influence β-arrestin mediated ERK activation [79]. When the receptor is activated, β-arrestin1 is recruited to the β2AR [48], where it is dephosphorylated at Ser 412; the SH3 domain of c-Src—Src kinase dephosphorylated at Tyr 530—associates with the Pro-X-X-Pro consensus sequence found in the N-terminal region of β-arrestin1; this complex facilitates Ras-dependent ERK phosphorylation and activation [21]. The resultant ERK pathway is important in cell survival, proliferation, and differentiation [48]. β-arrestin-mediated Src recruitment at the β2AR is also important in the brain endothelium, where Src phosphorylates cortactin, thereby facilitating fixation of meningococcus bacteria to endothelial cells [49]. Furthermore, β-arrestins are also involved in recruiting VE-cadherin and p120-catenin, which play a role in increasing the permeability of the endothelial cell barrier, aiding in meningococcal crossing of the blood-brain barrier [49]. However, a study using β-arrestin1/2 knockout cells and Gs knockout cells indicated that, following β2AR activation with isoproterenol and carvedilol, ERK phosphorylation was observed in cells lacking β-arrestin1 and 2 but not in cells lacking Gs, suggesting that G proteins, but not β-arrestin, are necessary in this pathway [48]. Addition of β-arrestin to β-arrestin1/2 knockout cells did produce stronger and longer-lasting ERK signaling, until excessive β-arrestin decreased ERK signaling [48]. This suggests that β-arrestins are functionally important in β2AR signaling as scaffolds, whereas G proteins may be necessary for the initiation of ERK activation [48].

### 3.7. Sphingosine-1-Phosphate Receptor 1

Sphingosine-1-phosphate receptor 1, also known as S1P1R is a high affinity receptor for the bioactive lipid (2S,3R,4E)-Sphingosine 1-phosphate [80,81]. Although the S1P1R expression is very abundant in endothelial cells, transcripts related to S1P1R are also detected at lower levels in vascular smooth muscle cells, fibroblasts, melanocytes, cells of epithelioid origin, brain [82], alveolar macrophages [83], cardiovascular tissues [84], natural killer cells and T cells [85]. S1P1 receptor is a G-protein coupled receptor which promotes signaling through Gαi/o proteins [86,87]. Endocytosis of G-protein-coupled receptor signal complexes is required for activation of ERK1/2. β-arrestin1 is recruited to ligand-bound S1P1R phosphorylated by GRK2. β-arrestin1 binds to Clathrin and activates Clathrin-mediated endocytosis [50]. Activation of ERK1/2 was shown to mediate (2S,3R,4E)-Sphingosine 1-phosphate-induced endothelial cell survival [51]. Prolonged desensitization of S1P1R via receptor phosphorylation, internalization, and degradation has been associated with endothelial cell damage and decreased survival [88,89,90].

### 3.8. Protease-Activated Receptor 1

Protease-Activated Receptor 1 (PAR1) contains a tethered ligand that is exposed after cleavage by thrombin and can increase vascular permeability [91]. Activated protein C (APC) also cleaves PAR1, resulting in exposure of a different tethered ligand with a distinct biological response [92,93,94]. Other groups have shown that APC binding to its receptor endothelial protein C receptor results in GRK5 recruitment to PAR1, which is crucial for promoting β-arrestin2 biased signaling by both APC and thrombin [95]. Despite their opposing effects, both APC and thrombin interact with PAR1 on endothelial cells [96]. A recent study demonstrated that APC activates β-arrestin-2, leading to PAR1-β-arrestin-2-MAPK 42/44 signaling that has a protective effect on endothelial function via PDGF-β [52]. APC enhanced tube-like capillary formation, migration, and transepithelial electrical resistance (TEER) while reducing permeability in bovine brain microvasculature endothelial cells [52]. In contrast, thrombin decreased expression of occludin, claudin-5, and ZO-1 and rapidly induced vascular permeability. Thrombin also reduced TEER rapidly while APC enhanced TEER more slowly. Consistent with bias between these two agonists, thrombin enhanced paracellular permeability while APC reduced paracellular permeability. Notably, the β-arrestin2 knockdown reduced while β-arrestin2 overexpression increased APC-induced tube-like capillary formation in HUVECs. The knockdown of β-arrestin2 also reduced migration, reduced TEER, and increased permeability in HUVECs [52].

### 3.9. Endothelin Receptors

Endothelin-1 (ET-1) was originally extracted from aortic endothelial cells and was identified as a strong vasoconstrictor [97] and to play an important role in the cardiovascular system. ET-1 and its related peptides, ET-2 and ET-3, exerts their biological effects through two types of receptors, type A (ET_A_R) and B (ET_B_R) [98]. ET_A_R is expressed primarily in smooth muscle cells and exerts vasoconstrictive and cardiotonic effects, while the ET_B_R is expressed primarily in endothelial cells and seems to antagonize ET_A_R signaling. ET-1 receptors are phosphorylated primarily via GRK2, however phosphorylation is not an absolute requirement for β-arrestin binding, desensitization, or internalization [99]. The knockdown of β-arrestin1 or β-arrestin2 in human kidney embryonic 293 cells resulted in enhanced ERK 1/2 phosphorylation in response to ET-1. A study shows that β-arrestins and EGFR transactivation are involved in ET_A/B_R signaling [57]. Epidermal growth factor receptors(EGFRs) were internalized in response to ET-1, while pretreatment with AG1478 (an EGFR antagonist) suppressed ERK1/2 phosphorylation in response to ET-1 [57]. Notably, β-arrestin1 has been shown to link ET_A_R to β-catenin signaling [53,54], Akt activation [55], and NFκB activation [56], consistent with it playing a central role in it promoting its pro-proliferative signaling.

### 3.10. Prostaglandin Receptors

GPCRs can also form functional homo- and heterodimers that act as distinct signaling hubs for cellular signal integration [100]. The AT1R and the prostaglandin F2α (PGF2α) receptor (FP), both of which are important in the control of smooth muscle contractility, have been shown to form functional heterodimeric complexes in HEK 293 and vascular smooth muscle cells [58]. Although FP does not normally recruit β-arrestin1/2 [58,97], as there are GPCRs that don’t recruit β-arrestin [101], stimulation of FP can recruit β-arrestin to an AT1R partner in the context of a heterodimer in both HEK 293 and vascular smooth muscle cells (VSMC) where they are endogenously co-expressed [58]. When the AT1R was treated with an antagonist, it strongly potentiated ERK1/2 activation by FP. In contrast, treatment of FP with an antagonists did not have a significant effect on AngII-mediated ERK1/2 activation [58]. Deletion of several Gα subunits significantly abrogated both AngII- and PGF2α-mediated β-arrestin1/2 recruitment which was in all cases restored by re-expression of Gα11, Gα12, or Gα13 but not always Gαq [102]. AngII induced a rapid, robust, and sustained recruitment of β-arrestin1/2 to AT1R and, to a lesser extent, the heterodimer, as expected, since AT1R is a strong recruiter of both β-arrestin subtypes [102]. However, PGF2α did not induce such recruitment to FP alone, although it did when the AT1R is present as a heterodimer [102]. Taken together, PGF2α specifically recruits and signals through β-arrestin but only in the context of the AT1R/FP dimer, suggesting that this may be a new allosteric signaling entity. Notably, a prostaglandin E2 receptor 4 (EP4)–β-arrestin signaling complex has been well characterized whereby GRK and protein kinase A (PKA) phosphorylation of EP4 recruits β-arrestin1 to activate c-Src to initiate EGFR transactivation. Activation leads to subsequent downstream signaling through PI3K and Akt [103]. It was recently recognized that the EP2 receptor regulates β-arrestin signaling to initiate PI3K/Akt, ERK, and c-Jun N-terminal kinase (JNK) pathways, which are particularly important for cell proliferation and migration [59,60].

## 4. β-Arrestin Regulation of Receptor Tyrosine Kinases

### 4.1. Vascular Endothelial Growth Factor Receptors

Vascular endothelial growth factor receptors (VEGFRs) belong to the family of receptor tyrosine kinases and play a central role in endothelial function, including cell proliferation and survival, angiogenesis, and lymphangiogenesis [104]. VEGFRs are activated by several vascular endothelial growth factors (VEGF-A, VEGF-B and VEGF-C) [105,106]. β-arrestin2 has been shown to regulate VEGF-mediated control of endothelial permeability through the endocytosis of VE-cadherin [107]. Knockdown of β-arrestin1 inhibited VEGF-C–induced human lung microvascular endothelial cells (HMVEC-L) proliferation, migration, and angiogenesis [108]. Consistent with that, knockdown of β-arrestin1 reduced VEGFR3, Akt, and endothelial nitric oxide synthase phosphorylation. This regulation was mediated by direct β-arrestin1 binding to the VEGFR3 kinase domain and resulted in decreased VEGFR3 internalization (Table 2) [108].

### 4.2. Epidermal Growth Factor Receptor

β-arrestins have been shown to exert antiapoptotic and cardioprotective effects through transactivation of epidermal growth factor (EGF) receptor (EGFR) via Src, matrix metalloproteinase, heparin-binding EGF-like growth factor (HB-EGF) [109]. β-arrestin-mediated pathway uses the β1-adrenergic receptor (β1AR) to transactivate the EGFR. β-adrenergic ligands that do not activate G protein signaling (i.e., β-blockers) alprenolol (Alp) and carvedilol (Car) induce β1AR-mediated transactivation of the EGFR and downstream ERK activation (Table 2) [110].

## 5. β-Arrestin Regulation of Receptor Serine-Threonine Kinases

### 5.1. Transforming Growth Factor-β (TGF-β) Receptor

The type III TGFβ receptor (TβRIII or betaglycan) is a coreceptor expressed in endothelial-specific heteromeric complexes [111]. β-arrestin2 binds and mediates its clathrin-dependent internalization, which downregulates both Smad phosphorylation and Smad-independent p38 phosphorylation [111]. Studies have also shown that β-arrestin2 mediates TβRIII-induced activation of Cdc42 and inhibits NF-kB -dependent cell migration [111]. Conversely, the interaction of β-arrestin2 with endoglin, an endothelial-specific TGFβ coreceptor involved in angiogenesis and the homeostasis of vessel walls, is responsible for sequestering ERK in the cytoplasm, which prevents cell migration without affecting Smad-dependent signaling (Table 2) [111].

### 5.2. Bone Morphogenetic Protein Receptors

Bone morphogenic receptors (BMPRs) are members of the transforming growth factor-superfamily and play a multifunctional role in aiding development that regulates cell proliferation, differentiation, and apoptosis in various tissue types [113]. The majority of ligands (BMP-2, BMP-4, BMP-6, BMP-7, GDF-5, and GDF-6) have high affinity for type I receptors, predominantly BMPRIA activin-like receptor kinase-3 (ALK3) or BMPRIB (ALK6), due to the formation of heteromeric receptor complexes [114]. Stimulation with these ligands induces angiogenesis, EC proliferation, and migration. BMPs have also been shown to play key roles in the inhibition of vascular SMCs, while enhancing the differentiation of these cells [113]. After BMP ligand induced heteromeric complex formation, type II receptor kinases phosphorylate the type I receptor, initiating intracellular signaling via activation of Smad proteins [113]. BMPRs can also initiate non-Smad signaling pathways involved in the pathogenesis of vascular diseases, such as MAPK, PI3K/Akt and protein kinase C (PKC) signaling pathways, and Rho-GTPases, which are potentiated by ALK6 through β-arrestin induced internalization [111]. A role for β-arrestin1 has been demonstrated in the regulation of BMPRII signaling through Smads and their transcriptional targets as reported in abstract for [112]; the manuscript describing these finding in detail is currently in preparation (S. Rajagopal, personal communication) (Table 2).

## 6. Potential for Targeting β-Arrestins and Endothelial Cells in Vascular Disease

Endothelial dysfunction, characterized by an imbalance in signaling between endothelium-derived vasodilators (eg, NO and prostacyclin) and vasoconstrictors (eg, thromboxane A2, ET-1, and AngII), has been associated with many cardiovascular diseases [115]. Endothelial dysfunction can occurs in diseases of large vessels, such as atherosclerosis, and of small vessels, such as PAH [116]. Targeting β-arrestin-mediated signaling by these receptors are predicted to have a number of potential benefits.

### 6.1. CXCR4

Several studies have cited CXCR4 signaling to be implicated in vascular diseases. Lung tissue and animal models for PAH have shown increased CXCR4 expression [117]. AMD3100, a known CXCR4 antagonist, attenuated pulmonary angiogenesis leading to prevention of hepatopulmonary syndrome (HPS) in mice [118]. Similarly CXCR4 knockdown models in HUVECs have shown potential to decrease angiogenesis suggesting abrogation of signaling within cancer tissues [63]. While no studies have directly shown β-arrestins to have therapeutic potential under CXCR4 signaling, its ability to inhibit CXCR4 signaling could lead to the development of novel therapies.

### 6.2. ACKR3

ACKR3 does not result in activation of signaling pathways typical of G proteins but does activate MAP kinases through β-arrestins. Injured coronary arteries from both humans and angioplasty wire injury mice exhibited endothelial ACKR3 expression. Conditional endothelial ACKR3 deletion promoted neointimal formation after endothelial injury and exacerbated heart functional impairment after myocardial infarction (MI). Both ACKR3 gene delivery via left ventricular injection and treatment with a CXCR7 agonist offered cardiac protection after MI [119]. ACKR3 is elevated in the endothelium of explanted human hypertensive lungs and circulating CXCL12 concentrations are significantly elevated in disease. ACKR3 inhibition by CCX771 or CCX773 blocked proliferation but not migration of human pulmonary microvascular endothelial cells in vitro. ACKR3 is the receptor through which endothelial cell regeneration and repair, and proliferation, is mediated, whereas signaling via CXCR4 is essential for chemotactic cell migration [110]. ACKR3 inhibitors will need to be tested in additional vascular disease models to see if targeting its signaling could be beneficial.

### 6.3. S1P1R

Current clinical molecules that have been developed to target S1P1R provide therapeutic benefits in autoimmune disorders given S1P1R’s role in lymphocyte trafficking and endothelial cell function. Given the deleterious effects of prolonged β-arrestin mediated desensitization of S1P1R in endothelial cell survival which pose, a recent study developed a G-protein biased compound, SAR247799 to see if the damaging side effects of S1P1R desensitization could be selectively inhibited [88]. SAR247799 was found to potently activate protection pathways under the S1P1R without causing receptor desensitization. Additionally the ligand improved microvascular response in a pig model of coronary endothelial damage as well as preserved renal structure and function in a rat model of renal ischemia

### 6.4. PAR1

Activation of PAR1 by thrombin and APC leads to paradoxical functions where thrombin promotes cell death and barrier disruption, while APC promotes cell protection and barrier protection [52]. Currently, PAR1 is the target of anti-thrombins such as dabigatran, which is known to prevent PAR1 cleavage, activation, internalization, and β-arrestin recruitment in vitro and can be used for the treatment of acute ischemic stroke and for preventing recurrence [120]. More recent studies have suggested β-arrestin biased signaling via APC at PAR1 as a novel avenue for therapeutics for treatment of stroke with a reduced risk of excessive bleeding. In particular, a Lys191-193 Ala APC mutant was found to decrease anticoagulant activity by >90% while retaining normal cell signaling while granting cardioprotection against ischemia [121]. Supporting this theory, a recent study found APC stimulated β-arrestin signaling at PAR1 provided protective effects in endothelial cells in mice after experiencing a stroke or high-fat diet induced obesity [52].

### 6.5. Endothelin Receptors

ET-1 is a strong vasoconstrictor that plays important roles in the pathogenesis and progression of cardiovascular remodeling. ET receptor antagonists (ERAs) are FDA-approved for the treatment of PAH by preventing vasoconstriction and abnormal smooth muscle cell proliferation [122]. Notably, the knockdown of β-arrestin1 or β-arrestin2 in human kidney embryonic 293 cells resulted in enhanced ERK1/2 phosphorylation and EGFR internalization in response to ET [110]. Pre-treatment with Ro318425 (a PKC inhibitor) or AG1478 (an EGFR antagonist) suppressed ERK1/2 phosphorylation in response to ET [57]. These findings suggest that both G proteins and β-arrestins contribute to abnormal cellular proliferation in PAH, and that ERAs may have a role in the treatment of other forms of vascular disease.

### 6.6. VEGFR

VEGFR signaling a common target for tumor angiogenesis [123,124]. Breast cancer mouse models have shown targeting β-arrestin1 regulates induction of VEGF-A via hypoxia-induced factor-1α. Use of anti-angiogenic drug, thalidomide, or imatinib mesylate, both induced nuclear export of β-arrestin1 to prevent VEGF transcription and release, suggesting targeting β-arrestin1 localization could be useful in inhibiting aberrant angiogenesis and metastasis [124].VEGFR3 was also recently found to be expressed in the endothelial tip cells during angiogenesis and in tumor vasculature [125,126]. VEGFRs are also implicated in PAH. In rodent models of PAH, the VEGFR2 inhibitor SU-5416 has been used in combination with chronic hypoxia to induce PAH [127]. Furthermore, endothelial cell–specific knockout of *Vefgr3* in mice causes exacerbation of chronic hypoxia-induced PAH, suggesting a role for VEGFR3 signaling in endothelial cells in the pathogenesis of PAH [128]. Recent studies have also linked β-arrestin1 with VEGR3 regulation in PAH. Both β-arrestin1 and VEGFR3 are reduced in human PAH and deletion of β-arrestin1 led to worse PAH and abrogation of VEGFR3 signaling in mice exposed to hypoxia [108]. Therefore, promoting β-arrestin-mediated VEGFR2 and 3 signaling may be useful in the treatment of PAH.

## 7. Conclusions

Endothelial cell dysfunction is a hallmark of several vascular diseases. Over a decade of research has implicated chemokine and other receptor signaling in endothelial cell physiology through their effects on growth, migration, and immune function. In this review, we highlighted various ways in which β-arrestin modulates key aspects of endothelial cell function through their regulation of GPCRs, RTKs and RS/TKs. At this time there have only been limited studies on how β-arrestins can act as nodes for integrating signaling from these different signaling axes. With additional studies that focus on such cross-talk and on the physiological implications of this signaling, we may develop novel insights into how targeting β-arrestin-mediated receptor signaling in the vasculature can be useful as a therapeutic strategy in vascular disease.

## Figures and Tables

**Table 1:** Summary of β-arrestin-mediated signaling at selected GPCRs in endothelial cells.

GPCR	β-Arrestin Modulated Signaling Activity	β-Arrestin Modulated Functional Response	Reference
CXCR3	Recruited to CXCR3 without ligand	Unclear	[35]
CXCR4	Receptor internalization, ERK activation	Provides protective effects during endothelial barrier impairment when heterodimerized with PAR1	[40,41]
ACKR3	ERK activation, Akt activation	Angiogenesis, Ca2+ mobilization, leukocyte extravasation	[33,34,42,43,44]
AT1R	ERK activation, Akt activation, Src recruitment	Proliferation of VSMCs	[45,46]
APJ	Receptor internalization	Hypertrophy	[47]
β2AR	ERK activation, Src recruitment, VE-cadherin recruitment, p120-catenin recruitment	Proliferation, permeability of the endothelial cell barrier	[21,48,49]
S1P1R	Receptor internalization	ERK activation, endothelial cell survival	[50,51]
PAR1	MAPK signaling	Enhances tube-like capillary formation, migration, TEER, and reduces permeability in microvasculature endothelial cells	[52]
ETR	Akt activation, NFκB activation, β-catenin signaling	EGFR transactivation, suppressed ERK1/2 phosphorylation, proliferation	[53,54,55,56,57]
FP	Recruit β-arrestin to AT1R in VSMC, ERK1/2 activation	Unclear	[58]
EP4	PI3K/Akt activation, ERK activation, JNK activation	cell proliferation and migration	[59,60]

**Table 2:** Summary of β-arrestin-mediated signaling at selected RTKs and RS/TKs in endothelial cells.

RTK or RS/TK	β-Arrestin Modulated Signaling Activity	β-Arrestin Modulated Functional Response	Reference
VEGFR	Akt activation, and endothelial nitric oxide synthase phosphorylation, VE-cadherin endocytosis	HMVEC-L proliferation, migration, and angiogenesis, cell permeability	[107,108]
EGFR	Src activation, matrix metalloproteinase, heparin-binding EGF-like growth factor (HB-EGF)	Anti-apoptotic and cardioprotective effects	[109,110]
TβRs	Clathrin-dependent internalization, downregulates both Smad phosphorylation and p38 phosphorylation	Inhibits NF-κB dependent cell migration	[111]
BMPRs	Smad signaling	Angiogenesis, proliferation, and migration.	[112]
